# Impact of Sepsis on High-Density Lipoprotein Metabolism

**DOI:** 10.3389/fcell.2021.795460

**Published:** 2022-01-05

**Authors:** Alexander C. Reisinger, Max Schuller, Harald Sourij, Julia T. Stadler, Gerald Hackl, Philipp Eller, Gunther Marsche

**Affiliations:** ^1^ Department of Internal Medicine, Intensive Care Unit, Medical University of Graz, Graz, Austria; ^2^ Department of Internal Medicine, Division of Nephrology, Medical University of Graz, Graz, Austria; ^3^ Department of Internal Medicine, Division of Endocrinology and Diabetology, Interdisciplinary Metabolic Medicine Trials Unit, Medical University of Graz, Graz, Austria; ^4^ Otto Loewi Research Center for Vascular Biology, Immunology and Inflammation, Division of Pharmacology, Medical University of Graz, Graz, Austria

**Keywords:** LCAT (lecithin-cholesterol acyltransferase) activity, CETP, PLTP, endothelial lipase (EL), lipoprotein, sepsis

## Abstract

**Background:** High-density lipoproteins (HDL) are thought to play a protective role in sepsis through several mechanisms, such as promotion of steroid synthesis, clearing bacterial toxins, protection of the endothelial barrier, and antioxidant/inflammatory activities. However, HDL levels decline rapidly during sepsis, but the contributing mechanisms are poorly understood.

**Methods/Aim:** In the present study, we investigated enzymes involved in lipoprotein metabolism in sepsis and non-sepsis patients admitted to the intensive care unit (ICU).

**Results:** In 53 ICU sepsis and 25 ICU non-sepsis patients, we observed significant differences in several enzymes involved in lipoprotein metabolism. Lecithin-cholesterol acyl transferase (LCAT) activity, LCAT concentration, and cholesteryl transfer protein (CETP) activity were significantly lower, whereas phospholipid transfer activity protein (PLTP) and endothelial lipase (EL) were significantly higher in sepsis patients compared to non-sepsis patients. In addition, serum amyloid A (SAA) levels were increased 10-fold in sepsis patients compared with non-sepsis patients. Furthermore, we found that LCAT activity was significantly associated with ICU and 28-day mortality whereas SAA levels, representing a strong inflammatory marker, did not associate with mortality outcomes.

**Conclusion:** We provide novel data on the rapid and robust changes in HDL metabolism during sepsis. Our results clearly highlight the critical role of specific metabolic pathways and enzymes in sepsis pathophysiology that may lead to novel therapeutics.

## Introduction

Sepsis accounts for a major proportion of intensive care patients and is a key factor for mortality and morbidity worldwide (Rhee et al., 2019; Rudd et al., 2020). The rates of sepsis have been increasing over the last decades, because of an ageing population and the more broadly adopted use of immunosuppressive therapies (Álvaro-Meca et al., 2018; McCreery et al., 2020). Currently, sepsis is defined by sepsis-3-criteria, which include a suspected infection by the treating physician and an increase in the sequential organ failure assessment (SOFA) score of at least 2 points (Singer et al., 2016). Because of the unreliability of clinical diagnosis, biomarkers are urgently needed to aid in the diagnosis and prognosis prediction of septic patients. Furthermore, new therapeutic targets and regimens based on biomarker risk stratification may improve patient outcome of this devastating disease.

Lipid profiles are commonly affected during sepsis (van Leeuwen et al., 2003; Lekkou et al., 2014; Cirstea et al., 2017). Both compositional changes, such as a rapid decrease of high-density lipoprotein cholesterol (HDL-C) levels and total cholesterol (TC) levels occur, as well as functional changes such as reduced arylesterase activity (AEA) of the high-density lipoprotein (HDL) associated paraoxonase 1 (PON1) or cholesterol efflux capacity (CEC). We were recently able to show that the AEA consistently predicted the ICU- and 28-day mortality of patients with sepsis and septic shock admitted to the ICU (Reisinger et al., 2020).

A few studies have been performed on lipoprotein metabolism in experimental endotoxemia or sepsis (Barlage et al., 2001; Levels et al., 2007; Trinder et al., 2019), but the mechanistic background of the altered HDL metabolism in sepsis as well as the differences between sepsis and critically ill ICU patients without sepsis are still poorly understood. Experimental studies have shown that the addition of lipopolysaccharides (LPS) to plasma results in a marked decrease in cholesterol ester transfer protein (CETP) activity, leading to changes in HDL levels that are considered an adaptive response to maintain or increase HDL-C (Masucci-Magoulas et al., 1995). A study assessing human genetics CETP gain-of-function cohorts and humanized mouse models found that inhibition of CETP may improve sepsis outcomes by maintaining HDL levels thereby modulating immunity (Trinder et al., 2021). CETP-inhibitors have mainly been investigated in other indications such as the treatment of hypercholesterolemia. A recent meta-analysis revealed that CETP inhibitors may reduce nonfatal myocardial infections or cardiovascular death, but the results did not reach statistical significance and the clinical relevance of these reductions is modest (Taheri et al., 2020).

HDL particles receive free cholesterol from the periphery, which is then esterified by lecithin-cholesterol acyltransferase (LCAT) to cholesteryl esters (CE) (Asztalos et al., 2005; Rousset et al., 2009). Another enzyme involved in lipoprotein metabolism is endothelial lipase, which is a driving factor for hydrolyzation of phospholipids (PL) of HDL particles. Furthermore, CETP transfers CE from HDL to low-density (LDL) and very low-density lipoproteins (VLDL) in exchange for triglycerides (TG), while the phospholipid transfer protein (PLTP) transfers PL from triglyceride-rich lipoproteins to HDL (Deckert et al., 2017; Trinder et al., 2019; Tanaka et al., 2020; Barker et al., 2021). PLTP may also transfer LPS from bacteria to HDL (Vesy et al., 2000). However, several other factors alter HDL composition and function under inflammatory conditions. The levels of the acute phase protein serum amyloid A (SAA) increase significantly during the acute phase response. SAA associates with HDL and alters HDL structure, function, and metabolism (Artl et al., 2000; Moya et al., 2012).

In the present study, we investigated which enzymes involved in lipoprotein metabolism are altered in sepsis and non-sepsis patients in the intensive care unit (ICU). In addition, we investigated if these enzymes exhibit a prognostic utility for ICU and 28-day mortality.

## Material and Methods

### Study Population and Study Design

We recruited adult patients (>18 years) with sepsis and septic shock as well as ICU controls admitted to the ICU of the Department of Internal Medicine at the Medical University of Graz, Austria, as previously published (Reisinger et al., 2020). In brief, sepsis patients were defined according to the sepsis-3 definition (Singer et al., 2016), while patients in the ICU control group had no sepsis or bacteremia at the time of sampling. The study protocol was approved by the Institutional Review Board (IRB) of the Medical University of Graz, Austria (30-258 ex 17/18) and complied with the Declaration of Helsinki. Written informed consent (IC) was obtained from all conscious participants, while in comatose non-survivors the need for written IC was waived by the IRB.

### Laboratory Analyses

Routine laboratory markers were measured in the central clinical laboratory of the Medical University of Graz using a Sysmex (Sysmex Austria GmbH), Cobas (Roche Diagnostics), or BN II analyzer (Siemens Healthcare) as appropriate. All other samples were contemporaneously measured after finishing recruitment in order to avoid any inter-assay variance.

CETP activity of serum was measured using a commercially available kit (ab196995, Abcam, Cambridge Science Park, Cambridge, United Kingdom), according to the manufacturer’s instructions. Specifically, the assay uses a donor molecule containing a fluorescent self-quenched neutral lipid that is transferred to an acceptor molecule in the presence of CETP. The CETP-mediated transfer of the fluorescent lipid to the acceptor molecule results in an increase in fluorescence intensity (excitation: 465 nm; emission: 535 nm). LCAT activity of serum was assessed by a commercially available kit (MAK107, Merck, Darmstadt, Germany) according to the manufacturer’s instructions. Serum samples were incubated with the LCAT substrate for 4 h at 37°C. The fluorescent substrate emits fluorescence at 470 nm. When the substrate is hydrolyzed by LCAT, a monomer is released that emits fluorescence at 390 nm. The LCAT activity is assessed over time and expressed in change of 470/390 nm emission intensity. We measured substrate turnover over 4 h and then calculated the corresponding substrate turnover per hour. LCAT protein concentration was assessed using a commercially available ELISA kit (RD191122200R, Biovendor, Brno, Czech Republic) and performed according to the manufacturer’s instructions. PLTP activity of serum was measured using a commercially available kit (MAK108, Sigma-Aldrich, PLTP Activity Assay Kit), according to the manufacturer’s instructions. The PLTP Activity Assay Kit includes proprietary substrates to detect PLTP mediated transfer of fluorescent substrate. Transfer activity results in increased fluorescent emission intensity (excitation: 465 nm; emission: 535 nm) from the assay. For SAA and EL measurements commercially available kits were used (KHA0012, SAA Invitrogen, Carlsbad, CA, USA, and Nr. 27182, EL full-length ELISA assay, IBL International GmbH, part of the Tecan Group, Hamburg, Germany); each measured in duplicates and handled according to the respective manufacturer’s instructions.

### Statistical Analyses

All statistical analyses were performed with SPSS 26 (SPSS Inc., Chicago, IL, United States) and Stata 15.0 (Stata Corp., Houston, TX, United States). Continuous variables were summarized as medians [25th–75th percentile], and categorical variables as absolute values and frequencies (%). Between-group differences were analyzed with cross-tabulations, Mann-Whitney-U-tests, χ^2^-tests, and Fisher’s exact tests, as appropriate. Correlations were computed with Spearman’s rank-based correlation coefficient. The prognostic associations between 28-day/ICU mortality and lipid parameters and other potential baseline predictors were quantified with univariable and multivariable logistic regression. Variables with a *p* ≤ 0.05 in univariable logistic regression were considered in the multivariable models. Significance level was defined at 0.05. Formal adjustment for multiple testing was not performed.

## Results

### Baseline Characteristics and Lipid Parameters of the Study Population

In our study, 53 patients in the sepsis and 25 patients in the control cohort were included, as previously reported (Reisinger et al., 2020). In brief, the median age of sepsis patients was 66 [50–75] years, and 40% of the sepsis cohort were female. 91% of the infections were community-acquired, and the most common focus was the lung (42%), followed by abdomen (17%) and urinary tract (11%). Median SOFA score was 9 [7–13] points and blood cultures were positive in 52% of patients. Baseline characteristics of sepsis ICU survivors and non-survivors were similar, and ICU- and 28-day mortality of the sepsis cohort were 36 and 47%, respectively.

The ICU control cohort consisted of patients without sepsis or bacteremia at the time of sampling acquisition and included patients with acute cardiovascular disease, cardiac arrest, intoxications, acute kidney injury and other conditions. Median age was 72 [65–79] years (*p* = 0.012 compared to sepsis cohort) consisting of 60% female patients, with similar rates of pre-existing diabetes or liver disease. Due to insufficient sample volume in the aliquot of one patient in the control cohort, only EL measurement but no further lipid metabolism analyses could be performed in this single patient.

The inflammatory markers were higher in sepsis patients compared to ICU controls without sepsis or bacteremia, including white blood count (WBC; 14.9 [9.1–26.5] vs. 9.1 [6.6–13.5] G/L, *p* = 0.011), C-reactive protein (CRP; 213 [119–309] vs. 12 [4–31] mg/L, *p* < 0.0001), procalcitonin (PCT; 8.8 [1.2–35.1] vs. 0.2 [0.1–0.3] ng/ml, *p* < 0.0001), and interleukin-6 (IL-6; 440 [146–1,333] vs. 35 [18–68] pg/mL, *p* < 0.0001). Sepsis patients without shock compared to those with septic shock, had a lower SOFA score at 8 [5–11] vs. 13 [8–14] points (*p* = 0.003). Furthermore, some inflammatory markers were higher in the septic shock group compared to the sepsis group including PCT (2.6 [0.4–34.2] vs. 18.8 [7.0–66.5], *p* = 0.011), and IL-6 (309 [127–628] vs. 658 [153–4,011], *p* = 0.027), but not WBC or CRP.

The lipoprotein profile was significantly different between ICU sepsis and ICU control patients. Levels of total cholesterol were similar (106 [84–130] vs. 114 [96–156] mg/dl, *p* = 0.193), but HDL-C levels (14 [7–33] vs. 39 [33–55] mg/dl, *p* < 0.0001) were significantly lower in sepsis compared to ICU controls (Table 1).

**TABLE 1:** Baseline characteristics and lipid parameters in the ICU sepsis cohort (*n* = 53) and ICU control cohort (*n* = 25). As partially reported previously in Reisinger et al. (2020). Data are reported as medians [25th–75th percentile], or absolute values and relative frequencies (%). Sepsis patients were ICU patients suffering from sepsis or septic shock. Controls were ICU patients without sepsis or bacteremia at the time of sample acquisition.

Variable	Sepsis cohort (*n* = 53)	Controls (*n* = 25)	*p*
Demographics			
Age (years)	66 [50–75]	72 [65–79]	0.012
Female sex	21 (40%)	15 (60%)	0.144
Quantitative lipid parameters			
HDL cholesterol (mg/dl)	14 [7–33]	39 [33–55]	<0.0001
Triglycerides (mg/dl)	162 [105–274]	115 [80–145]	0.006
Total cholesterol (mg/dl)	106 [84–130]	114 [96–156]	0.193
Inflammatory markers			
White blood count (G/L)	14.9 [9.1–26.5]	9.1 [6.6–13.5]	0.011
C-reactive protein (mg/L)	213 [119–309]	12 [4–31]	<0.0001
Procalcitonin (ng/ml)	8.8 [1.2–35.1]	0.15 [0.06–0.28]	<0.0001
Interleukin-6 (pg/mL)	440 [146–1,333]	35 [18–68]	<0.0001
Lipid metabolism markers			
Endothelial lipase (pg/ml)	300.0 [178.3–577.5]	123.2 [74.2–270.1]	0.001
SAA (µg/ml)	2,827 [917–6,852]	269 [34–344]^a^	<0.0001
LCAT activity (% substrate turnover per hour)	2.6 [2.0–3.1]	5.7 [4.5–6.6]^a^	<0.0001
LCAT concentration (µg/ml)	26.4 [21.2–31.8]	38.1 [28.3–45.3]^a^	<0.0001
CETP (pmol/h)	1.9 [0.2–4.4]	5.8 [4.1–8.8]^a^	<0.0001
PLTP (pmol/h)	8.5 [6.1–10.6]	5.0 [3.5–6.7]^a^	<0.0001
Sepsis severity and outcomes			
SOFA score (points)	9 [7–13]	5 [3–9]	<0.0001
28-day mortality	25 (47%)	4 (16%)	0.011
ICU mortality	19 (36%)	4 (16%)	0.110

a note that 1 patient in the control group had insufficient sample volume in the aliquot to perform lipid metabolism analyses.

Abbreviations: HDL, high-density lipoprotein; ICU, intensive care unit; SAA, serum amyloid A; LCAT, lecithin-cholesterol acyltransferase; CETP, cholesteryl ester transfer protein; PLTP, phospholipid transfer protein; N/A = not applicable, SOFA, sequential organ failure assessment.

**FIGURE 1:**
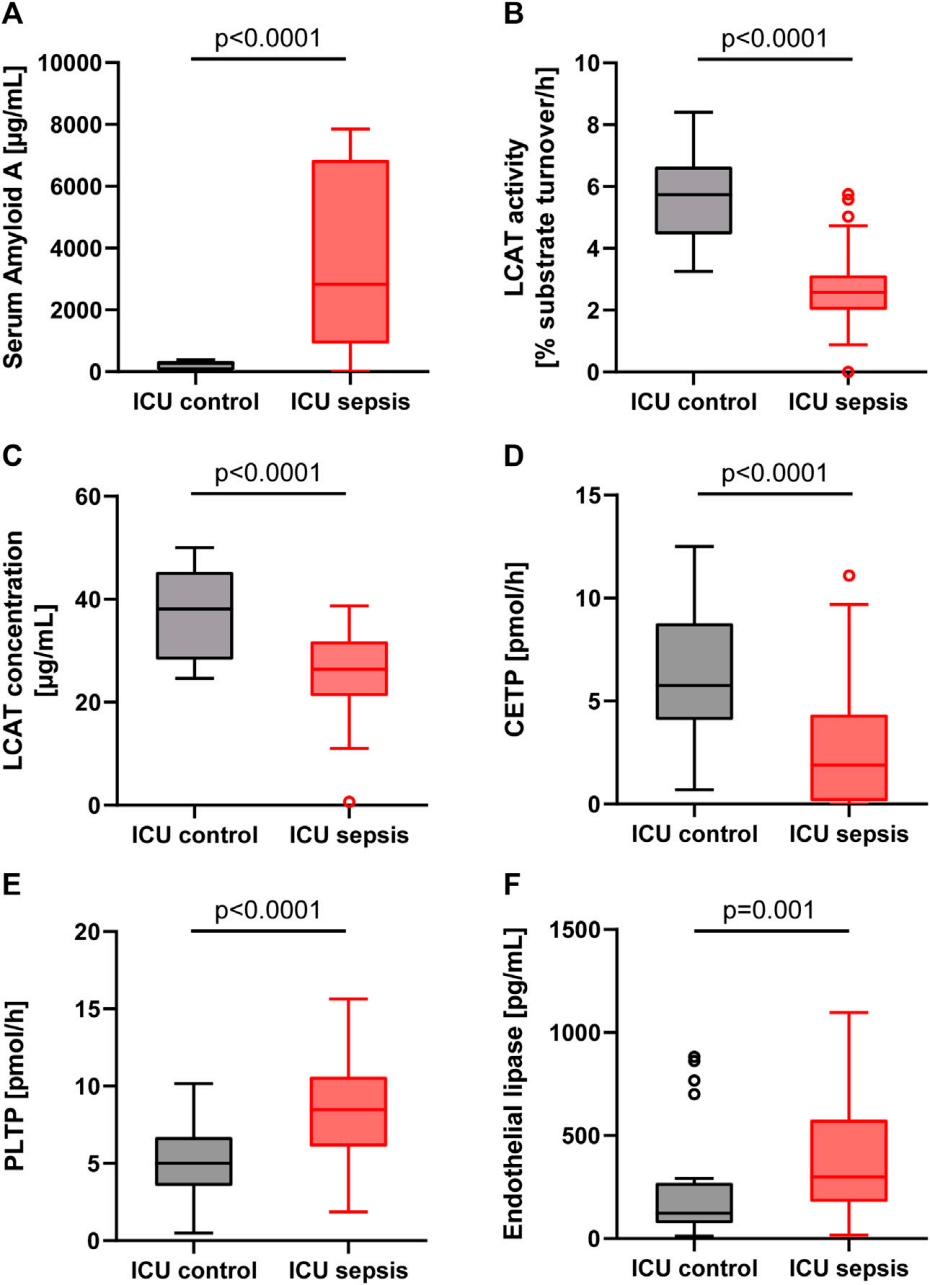
Boxplots for the ICU control group (patients without sepsis or bacteremia) and ICU sepsis group. **(A)**: Serum Amyloid A (SAA). **(B)**: Lecithin-cholesterol acyltransferase (LCAT) activity. **(C)**: Lecithin-cholesterol acyltransferase (LCAT) concentration. **(D)**: Cholesteryl ester transfer protein (CETP). **(E)**: Phospholipid transfer protein (PLTP). **(F)**: Endothelial lipase (EL). Note that in the EL boxplot two outliers in the sepsis group (17,436 and 1,659 pg/ml) and one outlier in the control group (2,839 pg/ml) are not displayed.

Levels of SAA were significantly higher in the sepsis group compared to ICU controls (2,827 [917–6,852] vs. 269 [34–344] µg/ml; *p* < 0.0001; Figure 1A). In the ICU control subgroup, SAA levels between ICU survivors at 248 [38–343] µg/ml and ICU non-survivors at 326 [22-n/a] µg/mL were similar (*p* = 0.742). Likewise, in the ICU sepsis subgroup, levels of SAA were not statistically significantly different between ICU survivors and non-survivors (3,897 [1,389–7,028] vs. 1,594 [337–5,880] µg/ml, *p* = 0.126; Table 2).

**TABLE 2:** Lipid metabolism markers in survivors and non-survivor in the sepsis cohort. Abbreviations: ICU, intensive care unit; LCAT, lecithin-cholesterol acyltransferase; CETP, cholesteryl ester transfer protein; PLTP, phospholipid transfer protein; SAA, serum amyloid A.

Variable	ICU survivors	ICU non-survivors	*p*
Endothelial lipase (pg/ml)	283.6 [163.9–451.1]	384.2 [201.1–661.7]	0.373
SAA (µg/ml)	3,897 [1,389–7,028]	1,594 [337–5,880]	0.126
LCAT activity (% substrate turnover per hour)	2.7 [2.3–3.4]	2.1 [1.6–2.8]	0.022
LCAT concentration (µg/ml)	26.8 [23.4–32.1]	24.4 [20.4–31.0]	0.243
CETP (pmol/h)	1.85 [0.00–4.53]	1.90 [0.30–4.10]	0.903
PLTP (pmol/h)	8.05 [5.86–10.21]	8.91 [6.32–11.92]	0.308

The LCAT substrate turnover, i.e., the LCAT activity, and the LCAT concentration were significantly lower in sepsis compared to controls (Figures 1B,C; Table 1). Sepsis survivors had higher levels of LCAT activity compared to sepsis non-survivors (2.7 [2.3–3.4] vs. 2.1 [1.6–2.8] %substrate turnover per hour; *p* = 0.022, Table 2), while controls had similar levels between survivors and non-survivors (6.0 [4.5–6.7] vs. 4.3 [4.1-n/a] % substrate turnover per hour; *p* = 0.172).

We investigated whether LCAT ratios, i.e. LCAT activity per hour divided by LCAT concentration multiplied by 100, were significantly different between groups. LCAT ratio was significantly lower in sepsis compared to control (10.0 [8.0–11.8] vs. 15.3 [13.3–17.6], *p* < 0.0001).

The activity of CETP was significantly lower in patients with sepsis compared to ICU controls (Figure 1D). However, there were no significant differences between ICU survivors and ICU non-survivors in the sepsis (1.85 [0.00–4.53] vs. 1.90 [0.30–4.10]; *p* = 0.903) or ICU control (6.1 [4.0–8.5] vs. 5.4 [4.3-n/a]; *p* = 0.805) group, nor any significant differences in patients without or with septic shock (1.40 [0.00–4.60] vs. 2.05 [0.53–4.10]; *p* = 0.802).

In comparison, the activity of PLTP was significantly higher in patients with sepsis compared to controls (8.5 [6.1–10.6] vs. 5.0 [3.5–6.7] pmol/h; *p* < 0.0001; Figure 1E), without significant differences between ICU sepsis survivors and non-survivors (8.05 [5.86–10.21] vs. 8.91 [6.32–11.92]; *p* = 0.308) or between ICU controls survivors at 4.99 [3.58–6.31] and non-survivors at 6.81 [3.20-n/a] (*p* = 0.561).

Levels of EL were significantly lower in controls compared to sepsis patients (Figure 1F; Table 1). Levels between survivors and non-survivors were not significantly different in either the sepsis (283.6 [163.9–451.1] vs. 384.2 [201.1–661.7]; *p* = 0.373) or control group (123.3 [76.7–496.0] vs. 95.6 [62.0–216.1]; *p* = 0.409).

### Correlations of Lipid Parameters in the Sepsis Group

The SOFA score showed an inverse correlation with HDL-C (rho = −0.308, *p* = 0.025) in the sepsis cohort, reflecting an HDL-C decline with increasing severity of organ dysfunction. The evaluation of enzyme activities revealed that LCAT activity was significantly inversely correlated with the inflammatory markers CRP (rho = −0.445, *p* = 0.001) and IL6 (rho = −0.440, *p* = 0.001), and positively correlated with LCAT concentration (rho = 0.494, *p* < 0.0001), HDL-C (rho = 0.446, *p* = 0.001), and CETP (rho = 0.297, *p* = 0.031). LCAT concentration, however, was not correlated with inflammatory markers. Correlation analyses of the other enzymes showed that PLTP correlated inversely with EL (rho = −0.322, *p* = 0.019), but no correlations with other lipoproteins, lipid metabolism or inflammatory parameters were found. In addition, no correlation with age or body mass index was found in this cohort. HDL-associated SAA levels correlated strongly with CRP (rho = 0.635, *p* < 0.0001), but not with enzymes involved in lipid metabolism, nor to other inflammatory markers.

### Lipid Parameters and Sepsis Mortality

The ICU- and 28-day mortality occurred in 19 (36%) and 25 (47%) of the 53 patients, respectively. In univariable logistic regression for 28-day mortality, age (OR per 5 years increase = 1.23 [1.02–1.50], *p* = 0.033), CRP (OR per 100 mg/L increase 1.72 [1.07–2.77], *p* = 0.025), and LCAT activity (OR per 1% increase in substrate turnover per hour 0.39 [0.19-0.77], *p* = 0.006) were significantly associated with outcome (Table 3). For 28-day mortality, LCAT prevailed in multivariable analyses; however, when including the SOFA score into the model LCAT was no longer significantly associated with outcome (Table 4). ICU mortality was determined by SOFA score (OR per 1 point increase 1.36 [1.12–1.65], *p* = 0.002) and LCAT activity (OR per 1% increase in substrate turnover per hour 0.51 [0.28–0.91], *p* = 0.023; Table 3). Similarly, in a multivariable model consisting of both variables, only SOFA score remained associated with outcome (Table 4). In sepsis patients, LCAT ratio was significantly associated with 28-day (OR per 1 increase 0.776 [0.637–0.946], *p* = 0.012) but not with ICU mortality (OR per 1 increase 0.864 [0.742–1.007], *p* = 0.062) in univariable logistic regression analysis. LCAT ratio did not remain significantly associated in multivariable logistic regression models. To investigate the role of LCAT in association with AEA, we performed a multivariable logistic regression using the above-mentioned variables with addition of AEA. In these models, LCAT was no longer significantly associated with ICU or 28-day mortality.

**TABLE 3:** Univariable logistic regression for ICU- and 28-day mortality in the sepsis cohort. Abbreviations: HDL, high density lipoprotein; ICU, intensive care unit; SOFA, sequential organ failure assessment; LCAT, lecithin-cholesterol acyltransferase; CETP, cholesteryl ester transfer protein; PLTP, phospholipid transfer protein.

Outcome variable	28-day mortality			ICU mortality		
Variable	Odds ratio	95% confidence intervall	*p*	Odds ratio	95% confidence intervall	*p*
Demographics						
Age (per 5 years increase)	1.23	1.02–1.50	0.033	1.06	0.89–1.27	0.511
Female sex	2.71	0.87–8.43	0.085	1.65	0.53–5.17	0.390
Quantitative lipid parameters						
HDL cholesterol (per 10 mg/dl increase)	0.88	0.66–1.18	0.381	0.84	0.61–1.17	0.312
Triglycerides (per 10 mg/dl increase)	0.98	0.93–1.04	0.501	0.99	0.93–1.05	0.715
Total cholesterol (per 10 mg/dl increase)	0.90	0.79–1.02	0.088	0.89	0.78–1.02	0.099
Laboratory covariables						
White blood count (per 1G/L increase)	1.02	0.98–1.07	0.357	1.00	0.96–1.05	0.960
C-reactive protein (per 100 mg/L increase)	1.72	1.07–2.77	0.025	1.40	0.90–2.18	0.136
Procalcitonin (per 1 ng/ml increase)	1.00	0.99–1.01	0.339	1.00	0.99–1.01	0.170
Interleukin-6 (per 100 pg/mL increase)	1.02	0.99–1.06	0.267	1.03	0.99–1.06	0.160
Lipid metabolism markers						
Endothelial lipase (per 100 pg/ml increase)	1.10	0.92–1.32	0.310	1.11	0.92–1.33	0.270
Serum amyloid A (per 1,000 μg/ml increase)	0.93	0.77–1.12	0.425	0.86	0.70–1.06	0.157
LCAT activity (per 1% increase in substrate turnover per hour)	0.39	0.19–0.77	0.006	0.51	0.28–0.91	0.023
LCAT concentration (per 1 μg/ml increase)	0.06	0.90–1.03	0.285	0.95	0.89–1.03	0.210
CETP (per 1 pmol/h increase)	0.94	0.78–1.14	0.542	0.98	0.80–1.19	0.811
PLTP (per 1 pmol/h increase)	1.04	0.88–1.23	0.641	1.10	0.92–1.31	0.306
Sepsis severity and outcomes						
SOFA score (per 1 point increase)	1.13	0.97–1.31	0.113	1.36	1.12–1.65	0.002

**TABLE 4:** Multivariable logistic regression for 28-day (A) and ICU- (B) mortality in the sepsis cohort.

Outcome variable			
Variable	Odds ratio	95% confidence intervall	p
A	28-day mortality		
Model #1			
LCAT (per 1% increase in substrate turnover per hour)	0.44	0.20–0.93	0.031
Age (per 5 years increase)	1.20	0.98–1.47	0.077
CRP (per 100 mg/dl increase)	1.22	0.70–2.21	0.493
Model #2			
LCAT (per 1% increase in substrate turnover per hour)	0.49	0.22–1.08	0.076
SOFA score (per 1 point increase)	1.13	0.93–1.36	0.212
Age (per 5 years increase)	1.25	0.99–1.58	0.051
CRP (per 100 mg/dl increase)	1.24	0.70–2.21	0.454

B	ICU mortality		
Model #1			
LCAT (per 1% increase in substrate turnover per hour)	0.59	0.31–1.13	0.111
SOFA score (per 1 point increase)	1.31	1.07–1.59	0.008

Variables with a *p* ≤ 0.05 in univariable logistic regression were considered in the multivariable models. An additional exploratory model with variables significant in univariable logistic regression and also including the SOFA, score was performed for 28-day mortality (model #2). Abbreviations: ICU, intensive care unit; SOFA, sequential organ failure assessment; LCAT, lecithin-cholesterol acyltransferase.

## Discussion

The central finding of our study was the uncovering of the manifold profound alterations in HDL metabolism that occur early after the development of sepsis. We observed marked differences in multiple enzymes involved in lipoprotein metabolism between ICU sepsis and ICU control patients. LCAT activity, LCAT concentration and CETP activity were significantly lower, while PLTP activity was significantly higher in ICU sepsis compared to ICU control patients. In addition, levels of SAA were increased 10-fold and EL levels were about 3-fold higher in sepsis patients compared to ICU controls. These changes involve enzymes produced by the liver such as LCAT, but also EL, which is derived from the vascular endothelium. The physiological role of LCAT is to esterify free cholesterol to cholesteryl-ester, after HDL received free cholesterol from the periphery (Rousset et al., 2009). Furthermore, LCAT shows antioxidative properties, and can hydrolyze oxidized phospholipids (Subramanian et al., 1999; Brites et al., 2017). Interestingly, we observed that LCAT activity was more than 50% decreased in sepsis patients compared to patients without sepsis or bacteremia. Familial LCAT deficiency leads to anemia due to accumulation of free cholesterol in red blood cells and to kidney failure. Recombinant LCAT may improve outcomes in these patients and may also provide a potential future drug in the armamentarium against sepsis (Shamburek et al., 2016a; Shamburek et al., 2016b; Ossoli et al., 2019). LCAT activity and concentration were correlated with HDL-phospholipid levels in our sepsis cohort. Furthermore, we found that LCAT activity was a significant predictor for ICU- and 28-day mortality. This effect remained significant even after including age and CRP in multivariable analyses in this study. However, when also considering the SOFA score as a marker for the severity of organ dysfunction, LCAT activity was no longer significantly associated with outcome. This finding may be explained by the sample size of our study, as the confidence interval shows a clear shift to the left of unity, and a larger cohort may have narrowed the CI leading to statistical significance. We furthermore did not find a correlation between LCAT and bilirubin in our study; and the decrease in LCAT activity is not solely explained by liver failure in sepsis patients. LCAT activity, but not LCAT concentration, was significantly inversely correlated with inflammatory markers such as CRP, PCT, and IL-6. Somewhat similar findings were shown in other studies, where LCAT activities were reduced during infection (Barlage et al., 2001; Levels et al., 2007). LCAT activity and concentration were furthermore strongly correlated with HDL-C in our study. This plausible finding is most likely due to a decrease in cholesteryl-ester formation and therefore lower HDL-C levels. In a study investigating HDL after endotoxin administration, LCAT and CETP were significantly decreased, and small/medium HDL particles were depleted (Moya et al., 2012). Furthermore, the free cholesterol to cholesteryl-ester ratio, which is determined by LCAT activity, was increased in septic patients in one study (Yamaguchi et al., 2018). Also, ApoA1 is partially replaced by SAA during acute inflammation, and ApoA1 is a main activator of LCAT (Meng et al., 1993). Likewise, median SAA levels in patients with sepsis were 2,827 μg/ml, whereas other critically ill ICU patients without sepsis or bacteremia had median levels of 269 μg/ml. However, we did not find an association of SAA levels, representing a strong inflammatory marker, with mortality outcomes in either group. Similarly no association of SAA with mortality was found for sepsis patients in another study (Cicarelli et al., 2008). This clearly underscores the importance of other metabolic pathways and enzymes that are decreased or increased during sepsis and that the outcome is not solely determined by inflammation itself. Both CETP and PLTP are part of the lipid-transfer and LPS-binding proteins (Hubacek et al., 1997; Kirschning et al., 1997; Levels et al., 2005). CETP can transfer CE *via* hydrophobic tunnels from HDL to LDL and VLDL, while PLTP transfers PL to HDL (Zhang et al., 2012; Barker et al., 2021). PLTP and CETP may protect against endotoxins and were considered to enhance LPS elimination (Kirschning et al., 1997; Cazita et al., 2008; Gautier et al., 2008). A decrease in CETP may additionally be regarded as an adaptive response by the human body trying to enhance HDL levels, but a relevant role of CETP for binding LPS is unlikely especially when compared to other enzymes such as lipopolysaccharide-binding protein (LBP) and bactericidal permeability increasing protein (BPI) (Clark et al., 2010; Dusuel et al., 2020). Furthermore, higher CETP expression in animal models lead to higher mortality in sepsis or endotoxemia (Dusuel et al., 2020). CETP activity was reduced in septic patients in our study compared to ICU controls. Similar results were found in a study from Llera Moya et al. (2012), who showed in healthy individuals that CETP activity decreases after endotoxemia was induced. In our study, we did find significant differences in CETP activity between ICU survivors or non-survivors. In good agreement, also, Grion et al. (2010) observed no difference in CETP levels on either day 0 nor day 3 (but only in the delta day 0–3) between survivors and non-survivors. PLTP on the other hand has strong binding properties for LPS (Hailman et al., 1996; Deckert et al., 2017). PLTP was increased during sepsis, and PLTP levels correlated with inflammatory markers (Levels et al., 2007). We similarly found that PLTP activity was significantly higher in sepsis patients at 6.5 pmol/h compared to controls at 5.0 pmol/h. However, we did not find correlations with inflammatory markers and levels were not significantly different between survivors and non-survivors, yet animal models showed that a substitution of recombinant human PLTP may improve sepsis outcomes (Deckert et al., 2017). Endothelial lipase is a phospholipase secreted mainly by vascular endothelial cells that is upregulated during inflammation (Miksztowicz et al., 2012; Yu et al., 2018). The main function of EL is the hydrolyzation of HDL-associated phospholipids (PL), which leads to increased turnover and alteration of HDL structure, and therefore to a decrease in HDL-cholesterol levels (Hirata et al., 1999; Jaye et al., 1999; Ishida et al., 2003). In endotoxemia studies, it was discovered that EL inversely correlated with HDL-PL levels (Badellino et al., 2008; Moya et al., 2012). In our study, we observed that EL levels are higher in patients with sepsis compared to ICU controls but did not find a correlation between HDL-PL and EL levels. Furthermore, we did not find significant correlations of EL with HDL-C levels suggesting a different mechanism for decreased HDL-C in sepsis, which is not solely explained by EL activity. EL was strongly correlated with IL-6, which is similar to a study from Badellino et al. (2008). To our knowledge, the findings of our study are the first report assessing EL in ICU sepsis patients. Badellino also found a correlation of EL with CRP, which we could not replicate in our study. However, the other group used high-sensitive CRP in asymptomatic subjects, whereas we used conventional CRP in ICU sepsis patients. Of particular interest, EL modification of HDL was recently shown to increase antioxidative capacity, to improve eNOS activating capacity and to modulate PON1 content of HDL (Schilcher et al., 2019; Radulović et al., 2020; Schilcher et al., 2021). These studies suggest that EL-modified HDL has improved vasoprotective properties.

Some limitations of our study have to be noted. Our investigation focuses on early changes in lipid metabolism and does not cover late time-points. Thus, the present data do not allow extrapolating for changes in a longer time range such as after treatment of sepsis in ICU after several weeks. Moreover, due to the small sample size, we cannot exclude the possibility that potential significant differences may not have been detected. Therefore, further studies in larger cohorts are warranted to confirm our results. Likewise, a control cohort with higher rates of organ dysfunction and more inflammation should be investigated in the future.

Taken together, we provide specific data on the rapid and robust changes of HDL metabolism in sepsis, in which HDL particles accumulate pro-inflammatory SAA on one hand, and lose HDL-C content and HDL-metabolism associated proteins such as LCAT on the other hand. Of particular interest, sepsis non-survivors had significantly lower LCAT activity compared to survivors. Furthermore, lipid transfer proteins such as PLTP and CETP are significantly altered during sepsis. These findings and the anti-inflammatory facets of HDL particles warrant further explorations as potential prognostic and therapeutic targets in septic patients.

## Data Availability

The raw data supporting the conclusion of this article will be made available by the authors, without undue reservation.

## References

[B1] Álvaro-Meca A. Jimenez-Sousa M. A. Jiménez-Sousa M. A. Micheloud D. Sánchez-Lopez A. Heredia-Rodríguez M. (2018). Epidemiological Trends of Sepsis in the Twenty-First century (2000-2013): an Analysis of Incidence, Mortality, and Associated Costs in Spain. Popul. Health Metrics 16 (1), 4. 10.1186/s12963-018-0160-x PMC5809921 29433513

[B2] Artl A. Marsche G. Lestavel S. Sattler W. Malle E. (2000). Role of Serum Amyloid A during Metabolism of Acute-phase HDL by Macrophages. Atvb 20 (3), 763–772. 10.1161/01.atv.20.3.763 10712402

[B3] Asztalos B. F. de la Llera-Moya M. Dallal G. E. Horvath K. V. Schaefer E. J. Rothblat G. H. (2005). Differential Effects of HDL Subpopulations on Cellular ABCA1- and SR-BI-Mediated Cholesterol Efflux. J. Lipid Res. 46 (10), 2246–2253. 10.1194/jlr.m500187-jlr200 16061948

[B4] Badellino K. O. Wolfe M. L. Reilly M. P. Rader D. J. (2008). Endothelial Lipase Is Increased *In Vivo* by Inflammation in Humans. Circulation 117 (5), 678–685. 10.1161/circulationaha.107.707349 18212282

[B5] Barker G. Leeuwenburgh C. Brusko T. Moldawer L. Reddy S. T. Guirgis F. W. (2021). Lipid and Lipoprotein Dysregulation in Sepsis: Clinical and Mechanistic Insights into Chronic Critical Illness. J. Clin. Med. 10 (8), 1693. 10.3390/jcm10081693 33920038 PMC8071007

[B6] Barlage S. Fröhlich D. Böttcher A. Jauhiainen M. Müller H. P. Noetzel F. (2001). ApoE-containing High Density Lipoproteins and Phospholipid Transfer Protein Activity Increase in Patients with a Systemic Inflammatory Response. J. Lipid Res. 42 (2), 281–290. 10.1016/s0022-2275(20)31690-4 11181759

[B7] Brites F. Martin M. Guillas I. Kontush A. (2017). Antioxidative Activity of High-Density Lipoprotein (HDL): Mechanistic Insights into Potential Clinical Benefit. BBA Clin. 8, 66–77. 10.1016/j.bbacli.2017.07.002 28936395 PMC5597817

[B8] Cazita P. M. Barbeiro D. F. Moretti A. I. S. Quintão E. C. R. Soriano F. G. (2008). Human Cholesteryl Ester Transfer Protein Expression Enhances the Mouse Survival Rate in an Experimental Systemic Inflammation Model: a Novel Role for CETP. Shock 30 (5), 590–595. 10.1097/shk.0b013e31816e30fd 18391856

[B9] Cicarelli D. D. Vieira J. E. Benseñor F. E. (2008). Comparison of C-Reactive Protein and Serum Amyloid a Protein in Septic Shock Patients. Mediators Inflamm. 2008, 631414. 10.1155/2008/631414 18385816 PMC2277077

[B10] Cirstea M. Walley K. R. Russell J. A. Brunham L. R. Genga K. R. Boyd J. H. (2017). Decreased High-Density Lipoprotein Cholesterol Level Is an Early Prognostic Marker for Organ Dysfunction and Death in Patients with Suspected Sepsis. J. Crit. Care 38, 289–294. 10.1016/j.jcrc.2016.11.041 28013095

[B11] Clark R. W. Cunningham D. Cong Y. Subashi T. A. Tkalcevic G. T. Lloyd D. B. (2010). Assessment of Cholesteryl Ester Transfer Protein Inhibitors for Interaction with Proteins Involved in the Immune Response to Infection. J. Lipid Res. 51 (5), 967–974. 10.1194/jlr.m002295 19965592 PMC2853464

[B12] Moya M. D. L. L. McGillicuddy F. C. Hinkle C. C. Byrne M. Joshi M. R. Nguyen V. (2012). Inflammation Modulates Human HDL Composition and Function *In Vivo* . Atherosclerosis 222 (2), 390–394. 10.1016/j.atherosclerosis.2012.02.032 22456230 PMC3361641

[B13] Deckert V. Lemaire S. Ripoll P.-J. de Barros J.-P. P. Labbé J. Borgne C. C.-L. (2017). Recombinant Human Plasma Phospholipid Transfer Protein (PLTP) to Prevent Bacterial Growth and to Treat Sepsis. Sci. Rep. 7 (1), 3053. 10.1038/s41598-017-03285-9 28596518 PMC5465182

[B14] Dusuel A. Deckert V. Pais de Barros J. P. van Dongen K. Choubley H. Charron E. (2020). Human CETP Lacks Lipopolysaccharide Transfer Activity, but Worsens Inflammation and Sepsis Outcomes in Mice. J. Lipid Res. 62, 100011. 10.1194/jlr.RA120000704 33500240 PMC7859855

[B15] Gautier T. Klein A. Deckert V. Desrumaux C. Ogier N. Sberna A.-L. (2008). Effect of Plasma Phospholipid Transfer Protein Deficiency on Lethal Endotoxemia in Mice. J. Biol. Chem. 283 (27), 18702–18710. 10.1074/jbc.m802802200 18458077

[B16] Grion C. M. C. Cardoso L. T. Q. Perazolo T. F. Garcia A. S. Barbosa D. S. Morimoto H. K. (2010). Lipoproteins and CETP Levels as Risk Factors for Severe Sepsis in Hospitalized Patients. Eur. J. Clin. Invest. 40 (4), 330–338. 10.1111/j.1365-2362.2010.02269.x 20486994

[B17] Hailman E. Albers J. J. Wolfbauer G. Tu A.-Y. Wright S. D. (1996). Neutralization and Transfer of Lipopolysaccharide by Phospholipid Transfer Protein. J. Biol. Chem. 271 (21), 12172–12178. 10.1074/jbc.271.21.12172 8647810

[B18] Hirata K.-i. Dichek H. L. Cioffi J. A. Choi S. Y. Leeper N. J. Quintana L. (1999). Cloning of a Unique Lipase from Endothelial Cells Extends the Lipase Gene Family. J. Biol. Chem. 274 (20), 14170–14175. 10.1074/jbc.274.20.14170 10318835

[B19] Hubacek J. A. Büchler C. Aslanidis C. Schmitz G. (1997). The Genomic Organization of the Genes for Human Lipopolysaccharide Binding Protein (LBP) and Bactericidal Permeability Increasing Protein (BPI) Is Highly Conserved. Biochem. biophysical Res. Commun. 236 (2), 427–430. 10.1006/bbrc.1997.6970 9240454

[B20] Ishida T. Choi S. Kundu R. K. Hirata K.-i. Rubin E. M. Cooper A. D. (2003). Endothelial Lipase Is a Major Determinant of HDL Level. J. Clin. Invest. 111 (3), 347–355. 10.1172/jci16306 12569160 PMC151857

[B21] Jaye M. Lynch K. J. Krawiec J. Marchadier D. Maugeais C. Doan K. (1999). A Novel Endothelial-Derived Lipase that Modulates HDL Metabolism. Nat. Genet. 21 (4), 424–428. 10.1038/7766 10192396

[B22] Kirschning C. J. Au-Young J. Lamping N. Reuter D. Pfeil D. Seilhamer J. J. (1997). Similar Organization of the Lipopolysaccharide-Binding Protein (LBP) and Phospholipid Transfer Protein (PLTP) Genes Suggests a Common Gene Family of Lipid-Binding Proteins. Genomics 46 (3), 416–425. 10.1006/geno.1997.5030 9441745

[B23] Lekkou A. Mouzaki A. Siagris D. Ravani I. Gogos C. A. (2014). Serum Lipid Profile, Cytokine Production, and Clinical Outcome in Patients with Severe Sepsis. J. Crit. Care 29 (5), 723–727. 10.1016/j.jcrc.2014.04.018 24891152

[B24] Levels J. H. M. Marquart J. A. Abraham P. R. van den Ende A. E. Molhuizen H. O. F. van Deventer S. J. H. (2005). Lipopolysaccharide Is Transferred from High-Density to Low-Density Lipoproteins by Lipopolysaccharide-Binding Protein and Phospholipid Transfer Protein. Infect. Immun. 73 (4), 2321–2326. 10.1128/iai.73.4.2321-2326.2005 15784577 PMC1087464

[B25] Levels J. H. M. Pajkrt D. Schultz M. Hoek F. J. van Tol A. Meijers J. C. M. (2007). Alterations in Lipoprotein Homeostasis during Human Experimental Endotoxemia and Clinical Sepsis. Biochim. Biophys. Acta (Bba) - Mol. Cel Biol. Lipids 1771 (12), 1429–1438. 10.1016/j.bbalip.2007.10.001 17980169

[B26] Masucci-Magoulas L. Moulin P. Jiang X. C. Richardson H. Walsh A. Breslow J. L. (1995). Decreased Cholesteryl Ester Transfer Protein (CETP) mRNA and Protein and Increased High Density Lipoprotein Following Lipopolysaccharide Administration in Human CETP Transgenic Mice. J. Clin. Invest. 95 (4), 1587–1594. 10.1172/jci117832 7706465 PMC295654

[B27] McCreery R. J. Florescu D. F. Kalil A. C. (2020). Sepsis in Immunocompromised Patients without Human Immunodeficiency Virus. J. Infect. Dis. 222 (Suppl. 2), S156–S65. 10.1093/infdis/jiaa320 32691837

[B28] Meng Q. H. Calabresi L. Fruchart J. C. Marcel Y. L. (1993). Apolipoprotein A-I Domains Involved in the Activation of Lecithin:cholesterol Acyltransferase. Importance of the central Domain. J. Biol. Chem. 268 (23), 16966–16973. 10.1016/s0021-9258(19)85288-2 7688720

[B29] Miksztowicz V. McCoy M. G. Schreier L. Cacciagiú L. Elbert A. Gonzalez A. I. (2012). Endothelial Lipase Activity Predicts High-Density Lipoprotein Catabolism in Hemodialysis. Atvb 32 (12), 3033–3040. 10.1161/atvbaha.112.300110 23104846

[B30] Ossoli A. Simonelli S. Varrenti M. Morici N. Oliva F. Stucchi M. (2019). Recombinant LCAT (Lecithin:Cholesterol Acyltransferase) Rescues Defective HDL (High-Density Lipoprotein)-Mediated Endothelial Protection in Acute Coronary Syndrome. Atvb 39 (5), 915–924. 10.1161/atvbaha.118.311987 30894011

[B31] Radulović S. Gottschalk B. Hörl G. Zardoya-Laguardia P. Schilcher I. Hallström S. (2020). Endothelial Lipase Increases eNOS Activating Capacity of High-Density Lipoprotein. Biochim. Biophys. Acta Mol. Cel Biol Lipids 1865 (4), 158612. 10.1016/j.bbalip.2020.158612 PMC7116681 31923467

[B32] Reisinger A. C. Schuller M. Holzer M. Stadler J. T. Hackl G. Posch F. (2020). Arylesterase Activity of HDL Associated Paraoxonase as a Potential Prognostic Marker in Patients with Sepsis and Septic Shock-A Prospective Pilot Study. Front. Med. 7, 579677. 10.3389/fmed.2020.579677 PMC7642222 33195328

[B33] Rhee C. Jones T. M. Hamad Y. Pande A. Varon J. O’Brien C. (2019). Prevalence, Underlying Causes, and Preventability of Sepsis-Associated Mortality in US Acute Care Hospitals. JAMA Netw. Open 2 (2), e187571. 10.1001/jamanetworkopen.2018.7571 30768188 PMC6484603

[B34] Rousset X. Vaisman B. Amar M. Sethi A. A. Remaley A. T. (2009). Lecithin: Cholesterol Acyltransferase - from Biochemistry to Role in Cardiovascular Disease. Curr. Opin. Endocrinol. Diabetes Obes. 16 (2), 163–171. 10.1097/med.0b013e328329233b 19306528 PMC2910390

[B35] Rudd K. E. Johnson S. C. Agesa K. M. Shackelford K. A. Tsoi D. Kievlan D. R. (2020). Global, Regional, and National Sepsis Incidence and Mortality, 1990-2017: Analysis for the Global Burden of Disease Study. The Lancet 395 (10219), 200–211. 10.1016/s0140-6736(19)32989-7 PMC6970225 31954465

[B36] Schilcher I. Stadler J. T. Lechleitner M. Hrzenjak A. Berghold A. Pregartner G. (2021). Endothelial Lipase Modulates Paraoxonase 1 Content and Arylesterase Activity of HDL. Int. J. Mol. Sci. 22 (2). 10.3390/ijms22020719 PMC7828365 33450841

[B37] Schilcher I. Ledinski G. Radulović S. Hallström S. Eichmann T. Madl T. (2019). Endothelial Lipase Increases Antioxidative Capacity of High-Density Lipoprotein. Biochim. Biophys. Acta (Bba) - Mol. Cel Biol. Lipids 1864 (10), 1363–1374. 10.1016/j.bbalip.2019.06.011 PMC6699986 31220617

[B38] Shamburek R. D. Bakker-Arkema R. Auerbach B. J. Krause B. R. Homan R. Amar M. J. (2016). Familial Lecithin:cholesterol Acyltransferase Deficiency: First-In-Human Treatment with Enzyme Replacement. J. Clin. Lipidol. 10 (2), 356–367. 10.1016/j.jacl.2015.12.007 27055967 PMC4826469

[B39] Shamburek R. D. Bakker-Arkema R. Shamburek A. M. Freeman L. A. Amar M. J. Auerbach B. (2016). Safety and Tolerability of ACP-501, a Recombinant Human Lecithin:Cholesterol Acyltransferase, in a Phase 1 Single-Dose Escalation Study. Circ. Res. 118 (1), 73–82. 10.1161/circresaha.115.306223 26628614 PMC4740220

[B40] Singer M. Deutschman C. S. Seymour C. W. Shankar-Hari M. Annane D. Bauer M. (2016). The Third International Consensus Definitions for Sepsis and Septic Shock (Sepsis-3). JAMA 315 (8), 801–810. 10.1001/jama.2016.0287 26903338 PMC4968574

[B41] Subramanian V. S. Goyal J. Miwa M. Sugatami J. Akiyama M. Liu M. (1999). Role of Lecithin-Cholesterol Acyltransferase in the Metabolism of Oxidized Phospholipids in Plasma: Studies with Platelet-Activating Factor-Acetyl Hydrolase-Deficient Plasma. Biochim. Biophys. Acta (Bba) - Mol. Cel Biol. Lipids 1439 (1), 95–109. 10.1016/s1388-1981(99)00072-4 10395969

[B42] Taheri H. Filion K. B. Windle S. B. Reynier P. Eisenberg M. J. (2020). Cholesteryl Ester Transfer Protein Inhibitors and Cardiovascular Outcomes: A Systematic Review and Meta-Analysis of Randomized Controlled Trials. Cardiology 145 (4), 236–250. 10.1159/000505365 32172237

[B43] Tanaka S. Couret D. Tran-Dinh A. Duranteau J. Montravers P. Schwendeman A. (2020). High-density Lipoproteins during Sepsis: from Bench to Bedside. Crit. Care 24 (1), 134. 10.1186/s13054-020-02860-3 32264946 PMC7140566

[B44] Trinder M. Genga K. R. Kong H. J. Blauw L. L. Lo C. Li X. (2019). Cholesteryl Ester Transfer Protein Influences High-Density Lipoprotein Levels and Survival in Sepsis. Am. J. Respir. Crit. Care Med. 199 (7), 854–862. 10.1164/rccm.201806-1157oc 30321485

[B45] Trinder M. Wang Y. Madsen C. M. Ponomarev T. Bohunek L. Daisely B. A. (2021). Inhibition of Cholesteryl Ester Transfer Protein Preserves High-Density Lipoprotein Cholesterol and Improves Survival in Sepsis. Circulation 143 (9), 921–934. 10.1161/circulationaha.120.048568 33228395

[B46] van Leeuwen H. J. Heezius E. C. J. M. Dallinga G. M. van Strijp J. A. G. Verhoef J. van Kessel K. P. M. (2003). Lipoprotein Metabolism in Patients with Severe Sepsis. Crit. Care Med. 31 (5), 1359–1366. 10.1097/01.ccm.0000059724.08290.51 12771603

[B47] Vesy C. J. Kitchens R. L. Wolfbauer G. Albers J. J. Munford R. S. (2000). Lipopolysaccharide-binding Protein and Phospholipid Transfer Protein Release Lipopolysaccharides from Gram-Negative Bacterial Membranes. Infect. Immun. 68 (5), 2410–2417. 10.1128/iai.68.5.2410-2417.2000 10768924 PMC97439

[B48] Yamaguchi J. Nagase M. Yamamoto Y. Sakurai A. Kubo A. Mitsuhashi H. (2018). Increased Oxidative Stress and Renal Injury in Patients with Sepsis. J. Clin. Biochem. Nutr. 63 (2), 137–143. 10.3164/jcbn.17-130 30279625 PMC6160724

[B49] Yu J. E. Han S. Y. Wolfson B. Zhou Q. (2018). The Role of Endothelial Lipase in Lipid Metabolism, Inflammation, and Cancer. Histol. Histopathol 33 (1), 1–10. 10.14670/HH-11-905 28540715 PMC5858721

[B50] Zhang L. Yan F. Zhang S. Lei D. Charles M. A. Cavigiolio G. (2012). Structural Basis of Transfer between Lipoproteins by Cholesteryl Ester Transfer Protein. Nat. Chem. Biol. 8 (4), 342–349. 10.1038/nchembio.796 22344176 PMC3792710

